# Emergence of Meningitis Caused by Nonvaccine Serotypes of Pneumococcus in Rural United States

**DOI:** 10.1155/2020/2372843

**Published:** 2020-11-27

**Authors:** Jessie Marks, Guru V. Bhoojhawon, Katherine D. Patrick, Kelly E. Wood

**Affiliations:** Stead Family Department of Pediatrics, University of Iowa Stead Family Children's Hospital, 200 Hawkins Dr., Iowa City, Iowa 52242, USA

## Abstract

Pneumococcal conjugate vaccines have decreased the rates of invasive pneumococcal disease (IPD) in children. Since vaccine introduction, however, rates of infection due to nonvaccine *Streptococcus pneumoniae* serotypes have increased. We now describe 3 meningitis cases due to the nonvaccine serotypes 35B and 11A from rural United States.

## 1. Introduction

Conjugate pneumococcal vaccines have significantly reduced the rates of invasive pneumococcal disease (IPD) due to focus on virulent serotypes [1, 2]. However, in recent years, several countries including the United States (U.S.) have reported an increased incidence of infections due to nonvaccine serotypes referred to as serotype replacement [3, 4].

We describe a U.S. case series of 3 vaccinated pediatric patients admitted over a 13-month time period in the postpneumococcal conjugate vaccine (PCV) 13 era, from December 2017 to December 2018, with meningitis due to the non-PCV-13 vaccine *Streptococcus pneumoniae* serotypes, 35B, and 11A/D. Therefore, nonvaccine serotypes are becoming the most common cause of meningitis in children in rural regions of central U.S., more common than meningococcal meningitis [5]. The catchment area for our children's hospital is the eastern one-half of Iowa and the far western region of Illinois.

Institutional review board approval was obtained.

## 2. Methods

Initial isolation of bacteria by culture was performed in the clinical microbiology laboratory at the University of Iowa Hospitals and Clinics (UIHC). In addition at UIHC, cerebrospinal fluid (CSF) polymerase chain reaction (PCR) testing was performed using the BioFire® FilmArray® meningitis/encephalitis (ME) panel which tests for 14 common bacterial, viral, and fungal pathogens. Pneumococcal serotype deduction was performed by PCR at the Minnesota Department of Health Laboratory (Saint Paul, Minnesota). The PCR assay did not differentiate between pneumococcal serotypes 11A and 11D [6].

### 2.1. Case Presentation

#### 2.1.1. Case 1

A 13-month-old immunized toddler with atopic dermatitis was brought to the emergency department (ED) with a 4-day history of fever, worsening somnolence, decreased oral intake, and vomiting. At presentation, he was afebrile, tachycardic, tachypneic, and hypertensive. On exam, he was lethargic without any focal deficits. He had received 4 doses of PCV13 according to schedule.

CSF analysis showed pleocytosis (221 white blood cells/millimeter [3] (WBC/mm^3^)) and hypoglycorrhachia. The meningitis/encephalitis panel was positive for *S. pneumoniae*. Blood and CSF cultures were positive for penicillin-resistant *S. pneumoniae* serotype 35B. Isolate had intermediate to ceftriaxone (minimum inhibitory concentration (MIC) 1 microgram/milliliter (mcg/mL)). Brain magnetic resonance imaging (MRI) with contrast showed changes consistent with meningitis.

His hospital stay was complicated by hemolytic uremic syndrome (HUS) (peak blood urea nitrogren (BUN), 93 milligram/deciliter (mg/dL), and creatinine, 2.4 mg/dL) requiring peritoneal dialysis and multiple transfusions and cytokine storm syndrome due to *S. pneumoniae* induced endothelial injury (ferritin, 3574 nanogram/milliliter (ng/mL)). He completed 14 days of intravenous (IV) ceftriaxone and vancomycin.

Immunologic evaluation was notable for a low total IgG (401 mg/dL; normal 453–916 mg/dL), lymphopenia (absolute lymphocyte count, 1376; normal, 3000–9500) with decreased CD3, CD4, CD8, CD56, and CD19 cells felt to be secondary to his critical illness and elevated total IgE (546 international units per milliliter (IU/mL); normal, <60 IU/mL) reflective of his atopic dermatitis. Random vaccine titers (IgG) were protective for PCV13 serotypes. Complement studies were normal. Spleen was present on imaging. After discharge, all abnormalities resolved on follow-up testing.

After discharge, auditory testing revealed bilateral profound sensorineural hearing loss requiring bilateral cochlear implants.

#### 2.1.2. Case 2

A 17-month-old immunized toddler with spastic quadriplegia, global developmental delay, and seizure disorder secondary to hypoxic ischemic encephalopathy at birth was brought to the ED with a 3-day history of nasal congestion, tactile fever, atypical seizure characteristics, and persistent somnolence. She was febrile and tachycardic. On exam, she was unresponsive with baseline hypertonic extremities. She had received 4 doses of PCV13 according to schedule.

Blood culture grew penicillin-sensitive *S. pneumoniae* serotype 11A/11D. CSF obtained after 2 days of antibiotic administration showed a pleocytosis (331 WBC/mm^3^), and the meningitis/encephalitis panel was positive for *S. pneumoniae*. CSF culture was sterile. Brain MRI with contrast showed baseline cortical atrophy with acute meningitic changes. She completed 14 days of IV ceftriaxone.

Immunologic evaluation was notable for low complement alternative pathway activity, with AH50 of 54% (normal ≥59%), attributed to acute illness with normal levels documented after discharge. Random vaccine titers (IgG) were protective for PCV13 serotypes. Quantitative immunoglobulin levels and lymphocyte subsets were normal. Spleen was present on imaging.

#### 2.1.3. Case 3

A 5-month-old, immunized former premature infant born at 33 weeks gestation was brought to the ED with 2 days of fever, irritability, and poor oral intake. He was febrile and hypertensive. On exam, he was listless and irritable when moved. He had received 2 doses of PCV13 according to schedule.

CSF analysis showed pleocytosis (1032 WBC/mm^3^), hypoglycorrhachia, and meningitis/encephalitis panel was positive for *S. pneumoniae*. Blood and CSF cultures were positive for penicillin-resistant *S. pneumoniae* serotype 35B. Isolate had intermediate to ceftriaxone (MIC, 1 mcg/mL). He completed 21 days of IV ceftriaxone and vancomycin due to persistent fever and elevated inflammatory markers.

Immunologic workup demonstrated a low quantitative IgG (133 mg/dL; normal, 232–1411 mg/dL) attributed to prematurity, acute illness, and physiologic nadir with documented normal levels on repeat testing after discharge. Complement levels and lymphocyte subsets were normal. Spleen was present on imaging.

He developed gross motor delay by the end of his hospital stay.

## 3. Discussion

This report from one of the more rural agricultural states in the U.S. contributes to prior surveillance data in the U.S. demonstrating that 35B and 11A are now amongst the commonest serotypes colonizing the nasopharynx [7, 8]. Our cases support a new shift with serotypes 11A and 35B emerging as newer causes of IPD in the post-PCV13 era, in the same way 19A did after PCV7 [8, 9]. Despite its previous association with a low likelihood of invasive disease, studies reveal 11A as an emerging cause of meningitis, as seen in one of our patients [1, 8]. Serotype 35B is particularly concerning given its frequent association with penicillin/ceftriaxone nonsusceptiblity, as in our patients, and increasing demonstration of multidrug resistance following the introduction of PCV13 [1, 10]. In Europe, serotype 35B has been associated with the highest risk of death due to IPD [10]. Although both of our patients with serotype 35B meningitis survived, one had severe complications, and both suffered long-term morbidity.

The increasing emergence of IPD cases caused by nonvaccine serotypes raises new questions about these entities, including their presumed lower virulence. As fewer historically virulent serotypes are reported, IPD cases continue to be reported along with changing antibiotic resistance patterns and serotype replacement [3]. The nature of IPD has shifted from the isolated bacteremia previously common to focal IPD such as meningitis or empyema, and higher hospitalization rates were seen prior to conjugate vaccination [11].

IPD disproportionately affects children under age two, in part due to higher nasopharyngeal colonization necessary for host invasion [1]. Studies have observed that IPD due to nonvaccine serotypes and/or those with presumed low invasive disease capacity such as 35B and 11A was more prevalent among children with comorbidities [8, 12]. In studies of children over 2 years without a predisposing condition, IPD may be a manifestation of an underlying primary immunodeficiency and warrant immune evaluation [13]. Our patients presented under 2 years of age and had abnormalities detected on immunologic evaluation. In all patients, one with low quantitative IgG and lymphopenia, one with isolated low quantitative IgG, and one with decreased complement alternative pathway activity levels normalized after recovery from illness. One patient was born prematurely, a known risk factor for IPD in the first 2 years of life, and had only received 2 doses of the PCV13 series [9, 14]. Studies have shown comparable immune response to a two-dose primary compared to three-dose primary series, except for serotypes 6B and 23F [15]. In one study, over 92% of infants (*n* = 258–264) achieved IgG level concentrations ≥0.35 micrograms/milliliter for the all vaccine serotypes, except 6B and 23F, after 2 doses of PCV13 [16].

A limitation of our report is that PCR testing did not differentiate between pneumococcal serotypes 11A and 11D. Since no cases of meningitis due to serotype 11D have been reported, we presume the isolate from case 2 was serotype 11A [17].

Finally, our 3 cases highlight the race against serotype replacement with each pneumococcal conjugate vaccine iteration. Prioritizing coverage for serotype 35B in particular deserves renewed emphasis given its demonstrated virulence, antibiotic resistance, and long-term sequelae.

## Data Availability

No data were used to support this study.
